# A Frailty Index from Next-of-Kin Data: A Cross-Sectional Analysis from the Mexican Health and Aging Study

**DOI:** 10.1155/2017/6069374

**Published:** 2017-04-19

**Authors:** Mario Ulises Pérez-Zepeda, Matteo Cesari, María Fernanda Carrillo-Vega, Guillermo Salinas-Escudero, Pamela Tella-Vega, Carmen García-Peña

**Affiliations:** ^1^Research Department, Instituto Nacional de Geriatría, Mexico City, Mexico; ^2^Gérontopôle, Centre Hospitalier Universitaire de Toulouse, Toulouse, France; ^3^Université de Toulouse III Paul Sabatier, Toulouse, France; ^4^Hospital Infantil de México Federico Gómez, Mexico City, Mexico

## Abstract

*Objectives.* To construct a frailty index from next-of-kin information of the last year of life of community-dwelling 50 years old or older adults and test its association with health services utilization.* Methods.* Cross-sectional analysis from next-of-kin data available from the last wave of the Mexican Health and Aging Study (MHAS).* Measurements.* Along with descriptive statistics, the frailty index (FI) was tested in regression models to assess its association with adverse outcomes previous to death: number of hospitalized days in the previous year and number of visits to a physician in the previous year, in unadjusted and adjusted models.* Results.* From a total of 2,649 individuals the mean of age was 74.8 (±11.4) and 56.3% (*n* = 1,183) were women. The mean of the FI was of 0.279 (±SD 0.131,* R* = 0.0–0.738) and distribution was biased to the right. There was a significant association (*p* < 0.001) between the FI and number of hospitalized days (*β* = 45.7, 95% CI 36.1–55.4,* p* < 0.001) and for the number of visits to a physician (*β* = 25.93, 95% CI 19.27–32.6,* p* < 0.001) both models adjusted for age and sex.* Conclusion.* The FI constructed with next-of-kin data showed similar characteristics to similar indexes of older adults. It was independently associated with health care use.

## 1. Introduction

Aging is a growing phenomenon all around the world. Along with this increase in the number of humans in the highest range of age, several problems come along [1]. Frailty is one of these problems, due to worsening of health condition of the frail older adult, having a higher proportion of adverse outcomes and elevated usage of health care. Moreover, it has been noted by previous research that a considerable number of older adults are frail in the last year of their lives [2–4]. However it is not common that health professionals routinely measure frailty, and some of the available datasets in order to study geriatric epidemiology lack appropriate measurements to operationalize frailty, rendering it invisible for quite a while [5]. On the other hand, the frailty index allows integrating an accurate measurement of frailty from different number of sources, even if these sources do not have frailty measurements [6]. Some works on this matter have shown that medical files—in particular from comprehensive geriatric assessments—could be used for this purpose (e.g., constructing a frailty index); however this information is difficult to obtain and in many cases restricted to physicians [7].

The frailty index has been shown to be useful in a number of different settings in order to assess frail older adults. For example, a recent study has shown how the frailty index (FI) can be used in the allocation of financial resources to nursing homes [8], pointing to the fact of the potential use of an FI for other purposes apart from health care. Moreover, indexes derived from the comprehensive geriatric assessment (CGA) have been also described and are derived from over three decades of the best available tool for assessing older adults in the clinical context, and as recently stated by Pilotto et al. in an extensive review of CGA it is the tool of choice to determine the clinical profile, risks, and prognosis of older adults [9]. The FI-CGA was aimed at generating an index that could be easily drawn from clinical data [10].

In addition to currently used sources to integrate a FI, relatives have been shown to provide reliable data in diverse settings as proxies; this approach has been intended also to integrate FI with accurate and reliable results, comparable to a regular FI; nevertheless this index was constructed by proxy of an alive older adult [11]. On the other hand, a frailty index constructed from information provided by family or related persons of a deceased older adult could give a clear picture on how the last months of life of the older adult were. Therefore the aim of this study is to construct a frailty index with data provided by next-of-kin of the last year of life of community-dwelling Mexican older adults from the Mexican Health and Aging Study (MHAS) and test its association with adverse health-related outcomes in order to assess its validity. Our hypothesis is that a FI constructed from next-of-kin information would be similar to those alive older adults with poor health and will have an independent association with the adverse outcomes.

## 2. Methods

We report data from the third wave of the MHAS (2012). MHAS is a cohort study that currently has four rounds, the last one in 2015, whose main objective is to analyze how Mexican adults age. Complete aims and description of this study are available elsewhere [12]. This round included a set of questionnaires that were answered by a member of the family of the deceased (i.e., the last year of life of a community-dwelling older adult).

Regarding FI, it was composed following the standardized procedure by Searle et al., which includes transforming each variable into a score of 0 (deficit absent) to 1 (deficit present) with possible intermediate scores [13]. All deficit scores were summed and then divided by 30 (total number of deficits in the current list) for each participant, with total scores for the FI ranging from 0 (no deficit present) to 1 (all deficits present). Deficits included in this index were from different domains: self-rated health, comorbidities, mental health, and somatic symptoms (see Table 1 for detail).

In order to test the hypothesis, adverse outcomes were defined as higher use of health services. The first one was the number of times the older adult visited a physician to his office (as an outpatient) for any reason. The second one was the number of times an older adult had overnight stays at a hospital.

In order to adjust for the years from death, differences between the years of death and the FI were tested. In addition, models were also adjusted for this variable. The proxy was always asked to remember the last year of life of the deceased, both for the FI items and the adverse outcomes, with questions beginning with “In the last year of life….”

The Institutional Review Boards or Ethics Committees of the University of Texas Medical Branch in the United States, the Instituto Nacional de Estadistica y Geografia, and the Instituto Nacional de Salud Pública in Mexico approved the study. The study adhered to the ethical guidelines of the Declaration of Helsinki and all next-of-kin signed informed consent, as well as all participants when study started.

## 3. Results

From a total of 2,743 40-year or older individuals (age at time of death) the mean of age was 74.8 (±11.4) and 56.3% (*n* = 1,183) were women. The mean of the 30-item frailty index (30i-FI) was of 0.279 (±SD 0.131), with a minimum of zero and a maximum of 0.738 (Table 1).

**Figure 1:**
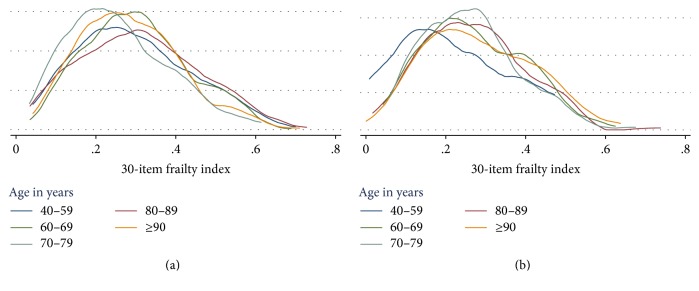
Kernel density plot of the 30-item next-of-kin frailty index by age category: (a) women and (b) men.

Distribution of the index was skewed to the right both for men and women; however it was more skewed for women as can be seen in Figure 1. In this same figure, the skewness is lower as the age gets higher also for both men and women (Figure 1).

There was a significant association (*p* < 0.001) between the index and number of hospitalized days with a beta coefficient of 46.04 (95% CI 36.5–55.5) and for the number of visits to a physician of 26.36 (95% CI 19.7–32.96) both models adjusted for age and sex. This means 5.3 more nights staying at the hospital for each standard deviation of FI or 7.75 more number of visits to a physician or for each standard deviation of FI (Table 2).

## 4. Discussion

As shown by our data a frailty index is feasible to be constructed from data provided by next-of-kin. Characteristics of the descriptive statistics and the distribution of the 30i-FI are similar to those reported in indexes constructed from other data sources, such as the higher burden of frailty in women [10, 14]. As expected, the distribution of the 30i-FI is biased to the right, which is characteristic of those older adults in the lower spectrum of health [14].

One of the main flaws of this work is the lack of objective measurements included in the index, which could lead to a memory bias coming from the proxy. On the other hand, frail older adults may have not visited as frequent as could have done it because of their vulnerability status, somehow underestimating the association with frailty. However, even that there was an observed underestimation of the index in those with more number of years deceased, when contrasting to health care use, adjusted models for this variable did not affect the estimates of the association. That is, a FI with data provided by next-of-kin could be useful to test economic analysis hypothesis such as cost analyses during the last period of life. On the other hand, our findings are amenable to be reproduced in other populations and having information from other sources, such as medical files. Some concerns arise when using parametric statistics with the frailty index, due to its biased distribution; however in this work the shifting towards the right allowed assuming a normal distribution and performing a linear regression.

Previous research in this cohort showed that a cut-off point of 0.21 in the frailty index was associated with higher mortality [15]. When looking at our results, the mean of the 30i-FI is above that cut-off point, which shows a highly deteriorated population, and as actually happened, with higher mortality. Also in Mexican older adults the index was found to be higher in women than in men and with a trend to bias to the right as age increases [16]; however these changes with sex and age categories were less evident in this report, in part due to the more homogeneous group; nevertheless still this phenomenon can be seen in the shown data. Careful interpretation should be made specially with those groups with a low number of subjects (i.e., ≥90 years old for men and women); because goodness-of-fit was low for these groups in the regression models (data not shown), further research should corroborate our findings with higher number of subjects in these groups. In addition, there is also scarce information on how frailty could be interpreted in younger adults, as shown in the first age category we presented (40–59); however some data point to the fact that frailty has a close relationship with age, even in younger subjects, since it is thought to be an accurate marker of biological age [17].

The appropriate stratification of older adults has been a concern long ago in geriatric medicine. This is particularly true when it comes to determine if an older adult is frail. A considerable number of tools have been developed to determine if an older adult is frail or not [18]. However, when it comes to stratification of risk in these age groups other tools have been also developed such as the multidimensional prognostic index that incorporates a number of variables of different domains and has shown accurate prediction of adverse outcomes [19]. Along with frailty tools, other stratification strategies should be tested in different contexts such as next-of-kin in order to have a higher number of options to assess older adults accurately.

## 5. Conclusion

The 30i-FI was gathered with information provided by a proxy and was independently associated with an increased use of health care in the last year of life of the elderly. This methodology could be used in other contexts and it could aid in the assessment of quality of care and health trajectories of terminal older adults.

## Figures and Tables

**Table 1:** General description of the sample including the variables of the frailty index.

Variable	Mean (SD) or *n* (%)
Age, mean (SD)	75.7 (10.7)
Age categories, *n* (%)	
40–59	184 (6.7)
60–69	640 (23.3)
70–79	849 (30.1)
80–89	777 (28.3)
≥90	293 (10.6)
Sex, *n* (%)	
Women	1,334 (48.6)
Men	1,409 (51.3)
The deceased had more weight with important medical decisions, *n* (%)	1,099 (40.1)
Years of death, median (IQR)	4 (0–11)
Number of hospitalized days in the last year of life, mean (SD)	12.6 (33.8)
Visited a physician in the last year of life, mean (SD)	11.6 (22.1)

Variables included in the index^*∗*^	

Self-rated health, *n* (%)	
Excellent	72 (2.6)
Very good	93 (3.3)
Good	717 (26.1)
Fair	989 (36)
Poor	872 (31.8)
Diabetes mellitus, *n* (%)	892 (33.6)
Cancer, *n* (%)	437 (16.5)
Lung disease, *n* (%)	405 (15.2)
Heart attack, *n* (%)	447 (16.8)
Stroke, *n* (%)	252 (9.5)
Hepatitis, *n* (%)	245 (9.2)
Tuberculosis, *n* (%)	21 (0.7)
Falls, *n* (%)	1,079 (40.7)
Fractures, *n* (%)	290 (10.9)
Pain, *n* (%)	1,368 (51.6)
Pneumonia, *n* (%)	245 (9.2)
Urosepsis, *n* (%)	475 (17.9)
Herpes, *n* (%)	67 (2.5)
Accident, *n* (%)	186 (7)
Dementia, *n* (%)	298 (11.2)
Memory complaint, *n* (%)	354 (13.3)
Incoherent conversations, *n* (%)	543 (20.5)
Frequently confused, *n* (%)	461 (17.4)
Bad judgment, *n* (%)	932 (35.1)
Depression, *n* (%)	1,150 (43.4)
Anorexia, *n* (%)	732 (27.6)
Urinary incontinence, *n* (%)	640 (24.1)
Weight loss, *n* (%)	1,314 (49.6)
Received help with certain activities, *n* (%)	1,491 (56.2)
Swelling feet/ankles, *n* (%)	1,140 (43)
Dyspnea, *n* (%)	815 (30.7)
Severe fatigue, *n* (%)	1,150 (43.4)
Coughing or wheezing, *n* (%)	894 (33.7)
Abdominal pain, *n* (%)	828 (31.2)
30i-FINoK, mean (SD)	0.279 (0.131)

30i-FINoK = 30-item frailty index from next-of-kin data.

^*∗*^Information is obtained from next-of-kin of the deceased regarding what happened in the last 12 months.

**Table 2:** Adjusted logistic regressions models ^*∗*^ with health care use as dependent and the 30-item frailty index from next-of-kin as the independent of interest stratified by age category and sex; totals for sex and for age category are also shown.

	Age category	Men		Women		Total^†^	
		OR (95% CI)	*p* value	OR (95% CI)	*p* value	OR (95% CI)	*p* value
Number of hospitalized days in the last year	40 to 59	3.14 (0.5–19.69)	0.221	6.61 (1.52–28.7)	0.011	4.69 (1.58–13.9)	0.005
	60 to 69	2.69 (1.21–5.96)	0.015	3.96 (1.92–8.16)	<0.001	3.3 (1.94–5.6)	<0.001
	70 to 79	1.65 (0.92–2.03)	0.08	3.41 (1.97–5.9)	<0.001	2.37 (1.6–3.51)	<0.001
	80 to 89	1.9 (1.21–3.27)	0.007	2.23 (1.37–3.63)	0.001	2.07 (1.46–2.92)	<0.001
	≥90	1.7 (0.75–3.87)	0.202	1.52 (0.75–3.09)	0.241	1.54 (0.93–2.71)	0.084
	Total	1.98 (1.46–2.68)	<0.001	2.63 (1.98–3.49)	<0.001	2.32 (1.89–2.85)	<0.001

Visited a physician in the last year	40 to 59	1.64 (1.15–2.32)	0.005	3.85 (0.83–17.8)	0.084	6.01 (1.43–25.1)	0.014
	60 to 69	5.45 (1.9–14.9)	0.001	4.94 (1.13–21.61)	0.034	5.2 (2.29–11.8)	<0.001
	70 to 79	2.71 (1.3–5.54)	0.006	3.08 (1.42–6.67)	0.004	2.84 (1.68–4.8)	<0.001
	80 to 89	2.95 (1.47–5.92)	0.002	1.5 (0.64–3.73)	0.321	2.32 (1.34–3.93)	0.002
	≥90	5.97 (1.7–20.95)	0.005	2.1 (0.85–5.24)	0.105	3.06 (1.5–6.26)	0.002
	Total	3.62 (2.42–5.43)	<0.001	2.52 (1.64–3.8)	<0.001	3.09 (2.31–4.15)	<0.001

^*∗*^Adjusted for age, more weight on medical decisions for the deceased, having medical services, and number of years since death of the older adult.

^†^Adjusted for the same variables above plus sex.
